# Patient with Recent Coronary Artery Stent Requiring Major Non Cardiac Surgery

**Published:** 2009-10

**Authors:** Usha Kiran, Neeti Makhija

**Affiliations:** 1 Prof and Head, Department of Cardiac Anaesthesia, Cardiac Neuro Center, All India Institute of Medical Sciences, New Delhi; 2 Assoc Prof., Department of Cardiac Anaesthesia, Cardiac Neuro Center, All India Institute of Medical Sciences, New Delhi,

## Abstract

**Summary:**

Anaesthesiologists are increasingly confronted with patients who had a recent coronary artery stent implantation and are on dual anti-platelet medication. Non cardiac surgery and most invasive procedures increase the risk of stent thrombosis especially when procedure is performed early after stent implantation. Anaesthesiologist faces the dilemma of stopping the antiplatelet therapy before surgery to avoid bleeding versus perioperative stent thrombosis. Individualized approach should be adopted with following precautions. i) In a surgical patient with a history of percutaneous coronary intervention (PCI) and coronary stent, determine the date of the procedure, the kind of the stent inserted and the possibility of complications during the procedure. ii) Consider all patents with a recent stent implantation (e.g. less than three months for bare metal stents and less than one year for brachytherapy or drug eluting stents as high risk and consult an interventional cardiologist. iii) Any decision to postpone surgery, continue, modify or discontinue antiplatelet regimes must involve the cardiologist, anaesthesiologist, surgeon, haematologist and the intensivist to balance the risk and benefit of each decision.

## Introduction

The incidence of coronary artery disease (CAD) has dramatically increased in India during the recent years to the extent that during the past 30 years the CAD rates have tremendously increased.^1^ The evolution of management of coronary artery disease (CAD) over the last 30 years has also been dramatic. Until 1960's, medical treatment was the mainstay of management. In 1970's coronary artery bypass grafting surgery (CABG) revolutionized coronary artery disease treatment, and the overall strategy became invasive. In 1980's, with advent of percutaneous transluminal coronary angioplasty (PTCA) and coronary artery stents in 1990's, there has been a shift towards less invasive modality for revascularization. Percutaneous coronary interventions (PCI) directed at severely stenotic lesions are highly effective in relieving angina. But a big question is whether this reduces the subsequent risk of major acute coronary events (MACE) such as death, myocardial infarction or angina?

Currently, over 90% of all PCI involve placement of stents^2^. Any PCI causes trauma to the vessel wall rendering the endoluminal surface thrombogenic. Hence adjunctive antiplatelet medication is crucial in preventing coronary thrombosis. Potential risk of non cardiac surgery shortly after coronary artery stenting prompted us to review the literature regarding optimal timing of safe surgery and challenges due to antiplatelet medication which concerns the anaesthesia team in decision making.

## Coronary stents

Revascularization with coronary balloon angioplasty may cause vessel spasm and abrupt closure due to vessel recoil. The deployment of stents after angioplasty reduces the risk of abrupt vessel closure by sealing coronary artery dissection. Stent deployment also reduces long term risk of restenosis by preventing elastic recoil and negative vessel remodeling.

Two major types of coronary artery stents are commonly deployed:

1. Bare Metal Stents (BMS)
2. Drug Eluting Stents (DES)

### Bare Metal Stents (BMS)

There is high incidence of late stent restenosis with BMS. In fact, restenosis is a side-effect of the normal healing process with the growth of the scar tissue around the stent mesh in a process called neointimal hyperplasia, which in some cases, can lead to occlusion of the coronary lumen. In patients receiving a BMS, in-stent restenosis requiring repeat intervention occurs in 12 – 20% of cases, especially it peaks at three months^3^. Preshibiterv et al also documented that BMS are associated with restenosis rate of 25 – 30%^4^ This process usually begins to occur in the first 6–8 weeks after stenting^5^ but can be seen beyond one year following stent placement.^6^ Procedures to treat stent restenosis are balloon angioplasty, mechanical de-bulking, repeat stenting, and intracoronary radiation brachytherapy).^7^

### Drug Eluting Stents (DES)

To prevent restenosis, drug eluting stents (DES) were designed, by coating a standard coronary stent with a thin polymer containing an antiproliferative substance that inhibits smooth muscle proliferation and neointimal hyperplasia within the stented segment.^8^ This has reduced the need for repeat intervention to almost 5%.^9^

Currently there are **two major types of DES** being inserted:

- Sirolimus Eluting Stents (‘Cypher’ stent) or
- Paclitaxel Eluting Stents (‘Taxus' stent).

**(SES) Sirolimus Eluting Stents:** Sirolimus **(rapamycin**) is a macrolide antibiotic with potent immunosuppressive and antimitotic properties, which binds to its cytosolic receptor FKBP 12, and inhibits down-regulation of cyclin dependent kinase inhibitor p27 ^kipl^. This blocks transition from G_1_ to S phase in the cell cycle and inhibits vascular smooth muscle cell proliferation and migration.^10^ Majority of the sirolimus is eluted from the polymer coating of the Cypher stent by 28 days^11^ and fully eluted in 60 days^12^ leaving a polymer BMS.

**(PES) Paclitaxel Eluting Stent**: Paclitaxel is a potent anti-tumor drug which inhibits microtubule formation during cell division.^8^ About 10% of the drug is released from the polymer coating of the Taxus stent by 10 days with the rest of the drug remaining within the polymer indefinitely.

DES has been shown to have as good safety profiles as BMS in the short to medium term (6 – 12 months). The mechanism of obstruction of DES is different from that of BMS. In DES the stent struts remain uncovered, hence prone to thrombosis. However in BMS, the pathophysiological mechanism of obstruction is restenosis with neointimal hyperplasia and DES inhibits this process.^13^ There are concerns that DES may cause endothelial dysfunction. This phenomenon may persist long after the drug is supposed to have fully eluted from the stent. There is a possibility of increase in the occurrence of acute infarction and late mortality with DES^14^

## Anti Platelet Therapy:

Antiplatelet therapy is mandatory for patients after coronary artery stenting as platelets play a major role in thrombus formation after coronary stenting. Coronary artery stents have been shown to be associated with a very high risk of thrombosis.^14^,^15^Clopidogrel combined with aspirin is the commonly prescribed regime. Another theinopyridine derivative, ticlopidine, can also be used. Therapeutic effects are similar with both of these drugs. However, ticlopidine is limited by side effects such as neutropenia and thrombocytopenia.^16^

**Mechanism of action** Clopidogrel and ticlopidine are metabolized to an active compound in the liver which inhibits P2Y 12 adenosine diphosphate (ADP) platelet receptor, thus inhibiting binding of fibrinogen to platelet glycoprotein IIb/IIIa receptor complex. This prevents platelet aggregation by ADP stimulation.^17^ Aspirin binds to enzyme cyclooxygenase (COX-1) and prevents conversion of arachidonic acid to thromboxane.

## When to start antiplatelet therapy

Clopidogrel therapy is initiated prior to or immediately following stenting procedure. A loading dose of clopidogrel 300 mg should preferably be given at least six hours prior to stenting procedure.^18^ This is followed by repeated doses of clopidogrel 75 mg per day. Repeated daily dose of 75 mg clopidogrel inhibits platelet aggregation with inhibition reaching a steady state between three to seven days following PCI.

## The most widely used antiplatelet regimes

**For Bare metal stents.** Loading dose of 300-600 mg of clopidogrel is given before implantation of a BMS. After the procedure aspirin, 75-100mg and clopidogrel 75mg are continued for 4-6 weeks. Low dose aspirin therapy is continued for life.

**For Drug eluting stents :** After the loading dose of 300-600mg of clopidogrel, 75 mg should be continued for a minimum of 3 months after implantation of sirolimuseluting stent and 6 months after a paclitaxel eluting stent. Aspirin dose and regime remain the same. Recent guidelines suggest dual antiplatelet therapy to be continued till one year.^19^–^21^

**Contraindications to clopidogrel**: Clopidogrel is contraindicated in patients with active pathological bleeding. Intracranial haemorrhage is an infrequent complication. Arare complication reported after clopidogrel was thrombotic thrombocytopenic purpura (TTP).^22^ Clopidogrel-induced platelet activation and aggregation was observed in this patient resulting instent thrombosis. However the incidence of TTP is very low.

### Resistance to antiplatelet medication

The potential risk of stent thrombosis in patients with coronary stents who experience resistance to antiplatelet medication must be considered in the perioperative setting.

**Clopidogrel resistance**: The term clopidogrel resistance encompasses patients for whom drug does not achieve its pharmacological effect. Failure of therapy reflects patients who have recurrent thrombotic events despite receiving therapy. Causes of clopidogrel resistance are listed in Table 1.

**Table 1:** Causes of clopidogrel resistance

**Causes of clopidogrel resistance are**:
a) genetic polymorphism of P2Y 12 receptor and CYP3A4
b) accrued release of ADP
c) Up regulation of other platelet activating pathways
d) Inter individual variability of platelet inhibition

No standard validated method is available to measure clopidogrel efficacy.^23^ Platelet function can be monitored by vasodilator-stimulated phosphoprotein, which directly measures the function of clopidogrel target P2Y 12 receptor. Platelet function can also be assessed by platelet aggregometry, flow cytometry of Pselectin, impedance aggregation, and platelet function analyser.^23^ Bleeding time is rarely used as it is highly operator dependent and poorly reproducible.^23^

There is inter individual variability of platelet inhibition by antiplatelet agents which may lead to clopidogrel resistance, as clopidogrel is a pro-drug, which requires activation by cytochrome 450 isoenzyme CYP3A4 24,25 and thereby response is variable (Table 2)

**Table 2:** Response to clopidogrel

**Response to clopidogrel**	
Non-response to clopidogrel	: If relative inhibition of ADP induced platelet aggregation of <10%
Response to clopidogrel	: If relative inhibition of ADP induced platelet aggregation of >30%
Low response	: In between the two

In a study by Aggarwal et al on platelet function using optical light aggregometry has shown that only 50% had a definitive response to clopidogrel.^26^ Grossmann et al has shown that at a median of 5 days after initiating clopidogrel treatment with a loading dose of 300 mg, only 0-5% patients receiving clopidogrel had an inadequate response^27^.

### Aspirin Resistance

Resistance to aspirin has also been described. Aspirin fails to reduce platelet production of thromboxane A_2_ by irreversible acetylation of cyclooxygenase-1 (COX -1) and thus fail to prevent platelet activation and aggregation.^28^ Aspirin resistance can be monitored by measuring platelet thromboxane production, or by assessing thromboxane–dependent platelet function. High urinary concentration of 11–dehydrothromboxane B_2_ a marker of aspirin resistance, has been shown to be associated with increased incidence of vascular events.^29^ A high dose of antiplatelet therapy is needed for patients who exhibit resistance,^30^ as impaired response to antiplatelet agent has been shown to enhance stent thrombosis.^31^

### Analyzing the risk of surgery after coronary artery stenting:

#### a) Risk of stent thrombosis

Kaluza et al first time in the year 2000 documented high risk of surgery in patients having recent insertion of coronary artery stents^32^. Out of 25 patients undergoing non-cardiac surgery with in 2 weeks of coronary stenting, 8 patients died. Out of which 6 deaths were due to acute myocardial infarction (AM1) and two because of bleeding. None of the patients who underwent surgery between 15 – 39 days after artery coronary stenting died.^32^ However Wilson et al reported mortality or AM1 or stent thrombosis in 8 out of 207 patients (3.9%) undergoing non-cardiac surgery within 2 months after receiving BMS^33^

Analyzing the outcome of the study undertaken by Sharma et al, on 27 patients who underwent non-cardiac surgery within 3 weeks after BMS implantation 6 out of 7 patients, in whom the thenopyridine was stopped for more than 5 days died compared to only 1 of 20 patients who continued thenopyridine therapy (p<0.001).^34^ Vicenzi et al reported 43% incidence of adverse cardiac events in 103 patients undergoing non-cardiac surgery after stent deployment.^35^ Reviewing the data about the risk of non-cardiac surgery in patients having DES deployed, McFadden et al reported stent thrombosis in 3 patients undergoing bladder polyp resection, colon cancer resection, and colonoscopy with polypectomy.^36^ Sirolimus eluting stent (SES) thrombosis was reported in 2 patients after surgery at 4 months and 21 months by Nesser et al.^37^

However in a single center series of 38 patients who underwent 41 major and 18 minor non cardiac surgeries at a median of 9 months after successful DES implantation no major adverse events were seen by Compton et al.^38^ No stent thrombosis was seen among 114 patients undergoing non-cardiac surgery after a median of 236 days from stent replacement by Bakhru et al.^39^ These studies explain the risk of surgery despite stent implantation in a patient of coronary artery disease, and highlights the importance of delaying surgery if and when possible following stent implantation.

Thromboelastogrpahy has shown evidence of hypercoagulability during surgery which may lasts for 7 days in post operative period, evident in the form of decrease reaction time (r-time), increase in clot strength, with continuous post operative increase in maximum amplitude (MA).^40^ Within 2 hours of completion of surgery MA has been shown to be increased by 68% raising the prediction of thrombotic complication.^41^ Hypercoagulability seems to be caused predominantly by platelet activity, which is not identified by standard coagulation monitoring^40^. There is a significant increase in ADP induced platelet aggregation at 24 – 28 h after surgery which persists till 7^th^ day. Platelets are not activated in the post operative period, but they are more prone to being activated as demonstrated by aggregation studies and the platelet count is significantly increased on 7^th^ day.^42^

Thus three factors play important role in acute stent thrombosis as shown in Table 3.

**Table 3:** Factors responsible for acute stent thrombosis

**Factor responsible for acute stent thrombosis**		
1.	Stopping protective antiplatelet therapy
2.	Hypercoaguable perioperative state
3.	Poorly endothelized stent

#### b) Antiplatelet therapy and risk of bleeding if antiplatelet medication is continued

Patients taking dual antiplatelet therapy may show increased risk of major bleeding complication. Increased bleeding time is seen when combined therapy of clopidogrel and aspirin is used, through synergistic antiplatelet actions.^43^ Whatever evidence of increased bleeding in the post operative period is available with combined therapy, it is in patients undergoing cardiac surgery.^44^–^45^ There are several papers describing series in which no difference was found in terms of bleeding between patients with and without dual antiplatelet therapy (aspirin plus clopidogrel). There is little evidence of increased surgical bleed in non-cardiac surgery. Rather no difference in the transfusion requirement was seen regardless of the use of antiplatelet agents by Sharma et al.^34^

#### c) Risk of coronary events if antiplatelet medication is discontinued

What is the overall risk of coronary adverse events in patients with stents if antiplatelet therapy is discontinued? There are no such reports which quantify this statement. There are few reports documenting adverse events when antiplatelet therapy was withdrawn. Kaluza et al showed that non-cardiac surgery soon after BMS placement was linked to a very high rate of adverse events.^32^ A case has been described where a patient with DES placement two weeks before surgery suffered a myocardial infarction in the post-anaesthesia care unit due to stent thrombosis. Patient had missed only one dose of aspirin and clopidogrel preoperatively.^46^

### AHA Guidelines following Coronary Artery Stenting

The American College of Cardiology (ACC), American Heart Association (AHA), European Society of Cardiology (ESC), Society for cardiovascular Angiography and Interventions (SCAI) and several other societies engage in production of guidelines in the area of cardiovascular diseases from time to time. These guidelines attempt to define the practices that meet the need of most patients in most circumstances. The aim of the guidelines is to improve the patient care. The ultimate judgment regarding the care of the particular patient is to be made by the clinician keeping in mind all the circumstances.

American College of Cardiology and American Heart Association (ACC/AHA) has laid down guidelines^18^ for antiplatelet therapy following coronary stenting, which were published in 2006. The guidelines have been updated from time to time.

### Important aspects of the guidelines are:

-A delay of 4-6 weeks between the BMS and elective non cardiac surgery is recommended to allow at least partial endothelization of the stent but not more than 12 weeks when restenosis may begin to occur.

-In patients treated with DES, elective surgical procedures with significant risk of bleeding should be deferred up to one year. In emergent non-cardiac surgery that requires stopping clopidogrel, the guidelines recommend continuing aspirin therapy if possible, and restarting clopidogrel as soon as possible.

-Risk of stopping the antiplatelet therapy should be weighed against the benefit of reduction of bleeding complication. If there is need to stop antiplatelet therapy, clopidogrel should be stopped for as minimum time as possible and restarted early. Aspirin should be continued perioperatively. Luckie et al^47^ in their recent article in 2009 have listed the following recommendations (Table 4)

**Table 4:** ACC/AHA Science advisory panel Recommendations

**: ACC/AHA Science advisory Panel Recommendations**
• Consider use of bare metal stents or balloon angioplasty rather than drugeluting stents in patients due to undergo non-cardiac surgery within 12 months.
• Healthcare providers to only discontinue antiplatelet therapy after discussion with the patients’ cardiologist.
• Patient education to ensure patients understand the need for continuous antiplatelet therapy and the risks of premature discontinuation.
• Postpone elective procedures with a significant bleeding risk for 12 months after stenting.
• For patients with DES where clopidogrel must b e discontinued, continue aspirin, restarting clopidogrel as soon as possible after the procedure.

**Approach to a patient for major surgery following recent coronary artery stenting** Regarding perioperative approach in patients with coronary stents there is no accepted standard or optimal approach for management.^48^ Multidisciplinary discussion between the cardiologist, surgeon, anaesthesiologist and haematologist should take place.

The key questions in such patients during pre anaesthesia evaluation are

1. When was the PCI done?
2. What is the type of stent?
3. How many stents were placed?
4. Was the revascularization complete?
5. Drug regime and any irregularities of the treatment?
6. History of any adverse cardiac event/stent thrombosis?
7. Urgency of surgery? / Can the surgery be delayed?
8. Bleeding risk during surgery?
9. History of conditions prone to stent thrombosis (Table 5)
10. Whether antiplatelet medication is to be maintained in perioperative period or stopped before operation?

**Table 5:** Stent thrombosis is commonly seen in

**Stent thrombosis is commonly seen in**		
1.	Small vessel
2.	Bifurcation lesion
3.	High risk patient as diabetes or renal failure
4.	Cessation of dual anti platelet therapy
5.	Sub optimal angiographic result

An important aspect of pre-operative visit is patient education. Patient should be explained the importance of antiplatelet therapy, the need to discontinue and to restart the therapy after surgery.

Investigations for platelet count and platelet function should be undertaken. Whole blood and platelet concentrates should be arranged prior to surgery.

**Fig 1:**
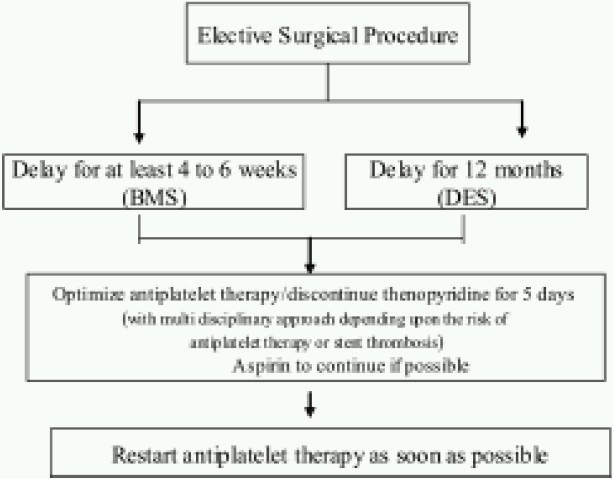
Algorithm for elective surgical procedures

**Elective surgical procedures**: It is the consensus that all elective procedures should be delayed for at least 4-6 weeks in patients who have received BMS. Preanaesthetic evaluation for elective surgery in patients with DES plays a significant role in decision making and risk assessment. Earlier reports suggested that if there is no major risk of bleeding, all elective surgeries within 6 months after DES implantation should be managed similar to urgent surgery. However the current consensus is that elective surgeries should be delayed for 12 months after DES placement (Fig 1).

### Urgent surgical procedures

**Fig 2:**
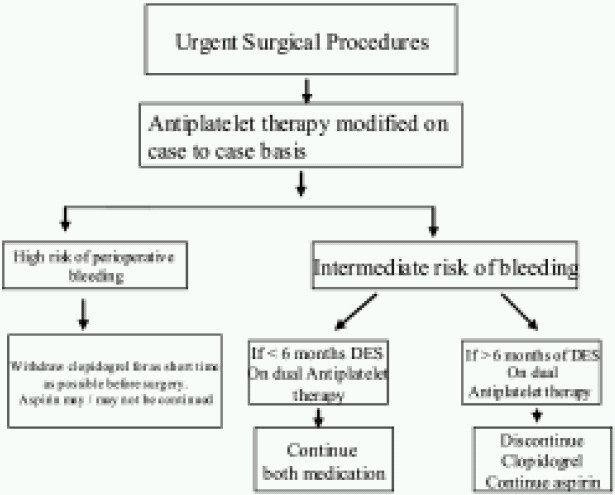
Algorithm for Urgent surgical procedures in patients with Drug eluting stent

For urgent surgical procedures, modification of antiplatelet medication should be individualized. When there is high risk of bleeding, as per latest guidelines, clopidogrel should be withheld for as short period as possible and restarted as soon as possible though the earlier recommendations were to stop for at least 5 days before surgery (Fig 2). Continuation of aspirin should be based on the nature of surgery. If there is intermediate risk of bleeding and the length of dual antiplatelet medication is less than 6 months, continue both medications; if more than 6 months, discontinue clopidogrel and continue aspirin.

In the operating room meticulous monitoring of electrocardiogram and trans esophageal echocardiography/trans thoracic echocardiography is essential for early detection of ischaemia/infarction, should stent thrombosis occur. In case of profound bleeding, platelet count and thromboelastography may be performed and need for platelet transfusion assessed.

An emergency bypass surgery or percutaneous intervention may be required in a patient with recent coronary artery stent undergoing major non-cardiace surgery. Hence the non cardiac surgery following recent coronary artery stent should preferably be performed in a centre where interventional cardiology care for prompt intervention is available, in case of stent thrombosis.

### Alternate anti-thrombotic therapy

An appropriate alternative anti-thrombotic strategy is a controversial issue, with no consensus of opinion for any particular regime. Heparin therapy either unfractionated by intravenous infusion or low molecular weight heparin by subcutaneous injection has been proposed during the period of time theinopyridine is stopped, but efficacy has not been proven.^35^

### Concepts about regional anaesthesia:

Dual antiplatelet therapy presents problems for regional anaesthesia. The placement of neuro-axial block in patients taking dual antiplatelet therapy cannot be recommended unless platelet function is with in acceptable limits or platelet transfusion is given before operation. The guidelines produced in 2003, by ASRA that without prior platelet transfusion clopidogrel should be stopped for minimum of 7 days and ticlopidine for a minimum of 14 days.^49^ The timing of the removal of epidural catheter and early reinstatement of the antiplatelet therapy must be considered. Delaying start of dual antiplatelet therapy in a patient of neuro-axial catheter removal may expose the patient to an unacceptable risk of stent thrombosis. Aspirin and NSAID do not represent an additional risk of spinal heamatoma, in patients receiving spinal or epidural anaesthesia.

### Prophylactic coronary revascularization

Prophylactic coronary revascularization with coronary artery bypass graft surgery (CABG) or PCI prior to non-cardiac surgery has previously been widely practiced, to reduce peri operative cardiac complications/adverse reactions in patients with known coronary artery disease. However, currently this practice is reduced as the data suggest that pre operative revascularization has little impact on peri operative adverse cardiac events when stenting is performed solely for this purpose.

If a patient with coronary artery disease is known to require non cardiac surgery, the first question to ask is whether the patient really needs revascularization. The CARP (Coronary Artery Revascularization Prophylaxis) study suggests that revascularization may not be necessary for a large number of patients without an unstable coronary syndrome or other high risk features. Pre operative prophylactic coronary revascularization should be considered as per ACC/AHA guidelines 50 (Table 6).

**Table 6:** Pre operative coronary Revascularization as per AHA/ACC guidelines.

1. Acute ST elevation M1
2. High-risk unstable angina or non-ST elevation myocardial infarction
3. Stable angina with left main stem stenosis
4. Stable angina with three-vessel coronary artery disease
5. Stable angina with two vessel disease involving proximal left anterior descending artery (LAD) and either LVEF < 50% and demonstrable ischaemia during non invasive testing

The risks and benefits of each strategy should be assessed, keeping in account patients symptoms, comorbidities, and coronary anatomy, degree of associated ischaemia and urgency and type of surgery to be performed.

### Future Prospects:

### Stents with pro-Healing Surfaces

Ongoing development in stent technology may render concerns regarding the long duration of anti-platelet therapy necessary following DES implantation obsolete. A number of different pro-healing surfaces are becoming available which may allow much more rapid and complete endothelialization of the stented segment. The Genous-R stent consists of a standard stainless steel stent, which is coated in a matrix containing monoclonal antibodies targeted specifically at the CD34 receptor. This receptor is exclusive to the surface of endothelial progenitor cells (EPC), which are preferentially captured onto the stent surface. Once attached to the stent surface, the EPCs mature into endothelial cells, rapidly creating a smooth endothelial surface within the stented segment without the risk of restenosis.^51^

### Conclusions:

One should learn from this review that patients with recent coronary artery stents are on antiplatelet therapy and are on high risk of peri-operative complication. The anaesthesiologist faces the dilemma of stopping the antiplatelet treatment before operation to avoid bleeding versus risk of post operative stent thrombosis. When faced with a patient who requires major non-cardiac surgery and has a coronary stent, the risk of stent thrombosis needs to be assessed against both the potential risk of bleeding and adverse consequences.

Till now there is no definite evidence that bleeding in non-cardiac surgery is not common and not a troublesome complication when compared with incidence of cardiac events. With high level of scientific evidence anaesthologist, surgeon and cardiologist should establish local treatment algorithms for the management of patients who have had previous PCI and coronary stent and are now to undergo surgery and follow the recommendations of current guidelines. Major noncardiac surgery should be avoided for 4-6 weeks for bare metal stents unless immediately life saving. After DES implantation, surgery within 3 – 6 months also substantially increases the risk of cardiac complications.

Non cardiac surgery should be delayed for 12 months. Even beyond 12 months, report of the late stent thrombosis suggest, that at least one antiplatelet agent should be continued perioperatively. Surgery which cannot be delayed should be performed on dual antiplatelet medication whenever possible

The multifaceted approach inclusive of cardiologist, surgeon, anaesthesiologist and haematologist should be used with each patient in order to provide maximum individual benefits. Each patient is different and treat the patient and not the stent.
